# Sanitation investments in Ghana: An ethnographic investigation of the role of tenure security, land ownership and livelihoods

**DOI:** 10.1186/s12889-016-3283-7

**Published:** 2016-07-18

**Authors:** Y. Awunyo-Akaba, J. Awunyo-Akaba, M. Gyapong, K. Senah, F. Konradsen, T. Rheinländer

**Affiliations:** School of Public Health, College of Health Sciences, University of Ghana, Legon, Accra Ghana; Dodowa Health Research Center, Ghana Health Services, Dodowa, Ghana; Future Generations International (FUGI), Ho, Ghana; Department of Sociology, University of Ghana, Legon, Accra Ghana; Department of Public Health, University of Copenhagen, Copenhagen, Denmark

**Keywords:** Ethnicity, Ghana, Land ownership, Livelihoods, Political power, Sanitation investments, Sanitation infrastructure

## Abstract

**Background:**

Ghana’s low investment in household sanitation is evident from the low rates of improved sanitation. This study analysed how land ownership, tenancy security and livelihood patterns are related to sanitation investments in three adjacent rural and peri-urban communities in a district close to Accra, Ghana’s capital.

**Methods:**

Qualitative data was gathered for this comparative ethnographic study over seven months, (June, 2011-January, 2012) using an average of 43 (bi-weekly) participant observation per community and 56 in-depth interviews. Detailed observational data from study communities were triangulated with multiple interview material and contextual knowledge on social structures, history of settlement, land use, livelihoods, and access to and perceptions about sanitation.

**Results:**

This study shows that the history of settlement and land ownership issues are highly correlated with people’s willingness and ability to invest in household sanitation across all communities. The status of being a stranger i.e. migrant in the area left some populations without rights over the land they occupied and with low incentives to invest in sanitation, while indigenous communities were challenged by the increasing appropriation of their land for commercial enterprises and for governmental development projects. Interview responses suggest that increasing migrant population and the high demand for housing in the face of limited available space has resulted in general unwillingness and inability to establish private sanitation facilities in the communities. The increasing population has also created high demand for cheap accommodation, pushing tenants to accept informal tenancy agreements that provided for poor sanitation facilities. In addition, poor knowledge of tenancy rights leaves tenants in no position to demand sanitation improvements and therefore landlords feel no obligation or motivation to provide and maintain domestic sanitation facilities.

**Conclusions:**

The study states that poor land rights, the history of settlements, in-migration and insecure tenancy are key components that are associated with local livelihoods and investments in private sanitation in rapidly changing rural and peri-urban communities of Ghana. Sanitation policy makers and programme managers must acknowledge that these profound local, ethnic and economic forces are shaping people’s abilities and motivations for sanitation investments.

## Background

### Access to adequate sanitation

Globally, governments and sanitation agencies are encouraging households to invest in and maintain their own household toilets with the different options available which meet international standards for ‘improved sanitation’. This refers to facilities and services that safely dispose of human excreta away from human contact and within the context of this study could also be termed as private or individual toilets. According to the World Health Organization (WHO), sanitation choices now considered unsafe include open defecation, communal and shared latrines, bucket and open pit latrines, latrines without slabs covering the opening and flush toilets that do not empty into a septic or sewer system [1, 2].

**Fig. 1:**
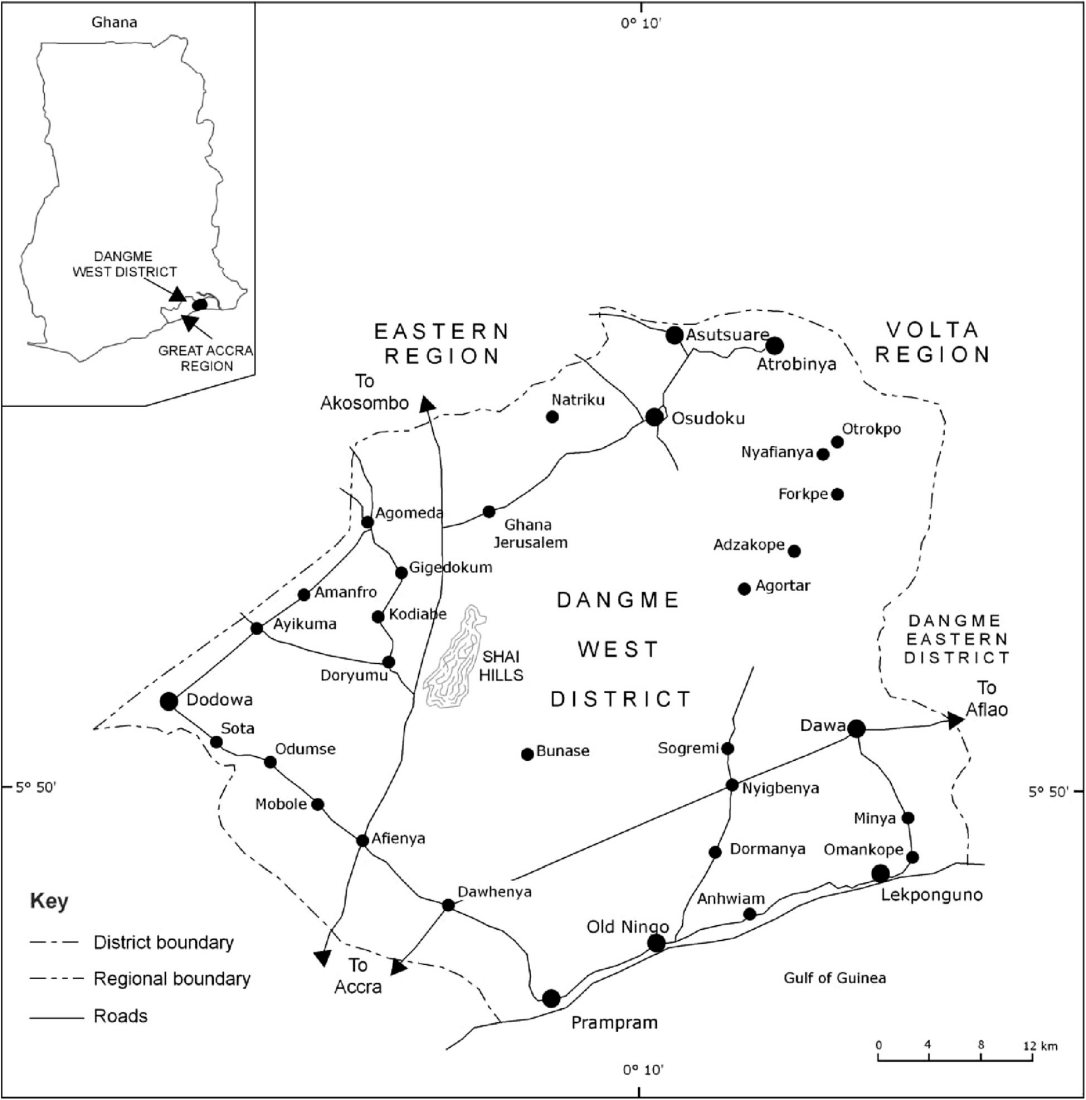
Shai Osudoku District Map (where Asutuare Area Council lies). Source: Shai Osudoku District (based on a map from Shai Osudoku District Assembly _ DODOWA) in [3]

With the deadline for reaching the 2015 Millennium Development Goals (MDGs) closing in, it is unlikely that Ghana will achieve the goal of halving the proportion of the population without access to improved sanitation (MDG 7). In 2015, only 15 % of Ghana’s population have access to improved sanitation, with a national rate of open defecation pegged at 19 %, but a much higher rate (34 %) for the rural population [3]. This situation is worrisome since improved sanitation is essential for health and well-being as well as healthy economic development [4, 5]. To achieve improved household sanitation coverage in Ghana, significant financial and infrastructural investments at the national, community and individual level are needed in addition to insights into the current barriers to uptake of sanitation.

### Barriers to household toilet ownership in Ghana

Sanitation studies have historically focused on understanding cultural and behavioural barriers to sanitation investments and ownership. Jenkins’ sanitation studies in Ghana and Benin thus stressed increased convenience, improved social status and prestige as important motivating factors for investments in household sanitation [6, 7]. Scott et al.’s study in Ghana found that sensory issues of smell, disgust and fear of diseases were driving hygiene behaviours and motivations for improved sanitation [8]. Several studies in Ghana also point towards economic factors as key barriers to private sanitation investment, including high costs of construction and lack of capital for investment [9, 10].

### Importance of land tenure considerations: security, land ownership and land rights for sanitation investments

Literature from Ghana indicates that issues of tenure security, land ownership and rights have significant implications for investments in improved livelihoods [11–13]. However, not much is known about how such issues may influence investments in sanitation.

In Ghana, land ownership has traditionally been perceived as not only including the earth or soil, but also the water and the properties situated on it [14]. Land is also believed to Land is also believed to play a religious or spiritual role, i.e. the abode and property of ancestors, the living and the unborn and the very source of life. Therefore, historically and presently, a large proportion of Ghanaian lands are held as communal property with indigenous people enjoying usufruct rights to the land through their chiefs [11, 15]. Fenske [11] undertook a comprehensive review of African land rights issues and concluded that traditional indigenous land distribution systems such as in Ghana may provide enough security for new investments and development. Others argue that promoting individual land titling to secure land rights is needed to increase investments in for example agriculture [12] and protect users against expropriation of land [13].

Studies in poor urban settlements in Ghana have indicated that uncertainty of land entitlement creates policy controversies and conflicts between local authorities and communities over the management of public toilets [16, 17]. In the absence of household toilets, 59 % of the population rely on shared toilets including public toilets, a barrier to individual toilet investments. The management of public toilets is also challenged by the poor maintenance of existing infrastructure, lack of transparent and rigorous regulation of the supervisors, and undue interference by political appointees. Other studies in urban Accra have highlighted that poor housing and tenure insecurity are central barriers for people’s willingness and ability to invest in environmental infrastructure and livelihoods [18–21]. Ghana’s 2010 revised Environmental Sanitation Policy’s recommendations for safe household latrines and adequate environmental sanitation within communities are based on the assumption of land tenure security for individuals [22]. Further, the Environmental Unit of the Ministry of Local Government and Rural Development as well as the Community Water and Sanitation Agency (CWSA) that together provide official sanitation oversight have no control over land availability or provision for building toilet facilities. We have little knowledge about how such issues of tenancy insecurity, landownership and land rights influence investments in sanitation, especially in areas undergoing rapid developments and changing livelihoods. This paper will analyse the influence of land ownership on sanitation investments in southern rural and peri-urban Ghanaian communities represented by three adjacent communities, with very different historical, economic and political roots and livelihoods.

## Methods

### Research approach

Seven months of ethnographic field work took place in two rural communities and one peri-urban community in the Shai Osudoku District in the Greater Accra Region of Ghana (Fig. 1) from June, 2011 to early January 2012. The study triangulated data from three case communities, 56 interviews, i.e. 21 for fishing; 25 farming; 10 estate, with different categories of community residents and leaders, and government representatives and detailed participant observations of community life. The source of the household and population estimates for the different communities was provided by the Dodowa Health Research Centre (DHRC). Their ongoing bi-annual Health and Demographic Surveillance System (HDSS) provided the demographic and background information for the three study communities [23].

The lead investigator and a female Ghanaian research assistant lived a short distance from the study communities for most of the study period. Where possible, they attended social events and related community activities. The primary investigator conducted all the fieldwork with interview and logistical assistance by the assistant who had prior fieldwork experience, and she was fluent in English and two languages spoken in the study area, Ewe and Twi. A Dangme (main language of the indigenes) speaking person living in the study area served as the liaison or contact between the study communities and the researchers. He had several years of work experience with the local Demographic Health Surveillance System [23] and thus had deep insights into the health status and development of the area.

#### Selection process

The selected study area was suitable for a comparative study because of the differences in the historical settlement and development, population make-up, and livelihoods represented by the three case communities of approximately the same geographical size. The differences in livelihoods were considered important because of variation in income levels, lifestyle choices and infrastructural development e.g. type of housing and sanitation investments. The livelihoods of each study community were influenced by the history of settlement, which also affected the population makeup. The contrasting characteristics offered the opportunity for investigating and comparing their influence on the prevailing sanitation behaviour and culture. The three communities can be described as fishing, rural-farming, and peri-urban (the latter also referred to as the ‘estate community’), respectively. The study area is also part of a local demographic and health surveillance system which provided detailed demographic and background information for this research. Case communities were also chosen since they were accessible by foot or public transport from the main town. A transect walk was conducted through each of the 3 study communities with the contact person and one or two other residents for a community overview. This tool depicts a systematic walk through the study area to find out the location, distribution and characteristics of its resources, and facilities [24]. This was followed by community familiarization walks over the 3–4 weeks within all the communities [25] to gain an in-depth knowledge of the main sanitary facilities e.g. pit latrines and Ventilated Improved Pit (VIP) toilets, the focal sources of domestic water, main land uses, infrastructural systems, and resources. Community maps were drawn together with photographs of key features that illustrated important landmarks and features [26].

#### General community observations

Based on sequential visits to the communities i.e. 48 – fishing; 41 – farming; 42 – estate over the study period, community profiles were developed, including details on the socio-demographics of their constituents, the history of the settlements, core infrastructures, the occupation and migration characteristics, public institutions, general hygienic and sanitary conditions and common family and housing structures. Throughout the study period, the primary investigator specifically visited community water and sanitation facilities to observe their usage and maintenance conditions, and to conduct informal interviews with encountered community members about facilities. The participant observational research involved normal involvement in the homecare and occupational activities related to the study participants where possible during community and home visits. These observational studies also involved visits to strategic occupational sites including rice farms, banana farms and fishing trips to observe the livelihood operations of study respondents. Informal and spontaneous discussions were video recorded with permission and later written down as observational notes. This data further included hand drawn maps showing the major landmarks within each community and detailed descriptions of interesting events observed. Since these observations took place along the interviews, they helped clarify questions, and puzzles arising from the interviews based on the regular reflection on the data collection. We sought further explanations on unexplained observations through the probes included in the in-depth interviews and FGDs. This was useful in helping to progressively answer the issues raised by the study and served as a way of reaching the saturation level.

#### Household observations

Twenty-one households were purposively selected for in-depth studies within the case communities (6–8 households per community). Chosen households presented a variety of sanitation and hygiene conditions (e.g. availability or absence of a latrine, drainage and garbage systems). Also, households’ access to water connections and sources, the variety of occupations, settlement and migration pattern and sources of income, provided diversity for the study. Participant observations were conducted of the people’s daily routines with a specific focus on gaining insights into occupational activities as well as domestic and personal hygiene practices.

#### Qualitative interviews

The tools for the study were developed in English and translated into Ewe and pre-tested in one of the potential study communities to access the understandability and ease of use of the guides [27]. Twenty five semi-structured interviews were conducted with residents of the three communities and local business owners about the history of settlement, social structures and general water, hygiene, sanitation, and health conditions of communities. These respondents were selected using the snowball [28] method (asking study participants to refer other potential respondents) following initial recommendations by the study’s community contact person. The first group of respondents consisted of 1–3 residents per community who further referred us to the second group (up to 4 members per community) to corroborate and offer additional information. This second group then referred us to a third group of community members who were also interviewed. No additional referrals were sought when the people being recommended by respondents’ had already being interviewed and the information the respondents’ offered was not new but only collaborated previous data.

Further, 31 semi-structured interviews were carried out with community leaders and government representatives (school teachers, local health personnel, environmental officials, local government officials and traditional leaders). The interview respondents were selected either through initial recommendation from the HDSS field contact person, by the researchers during familiarization visits and personal interactions based on resident or leader’s knowledge about the issues under investigation or from referrals from respondents about others who could provide additional information on specific issues. These interviews provided a further in-depth understanding of community challenges to sanitation development, occupation and housing. Observed practices deemed interesting were noted as field notes, or photographed and recorded as video clips. The observational data was used together with the interview data to answer queries and conflicting responses in a fluid mode to provide further insights until no new information was learned. None of the participants we selected for the household and qualitative interviews declined to participate even though some were more welcoming and open than others. The favourable response from the participant families could be due to the familiarization and introductory visits that occurred prior to recruiting them for the study.

#### Data management, processing and analysis

All interviews lasted between one and two hours and were conducted with the participant’s home or place of work with no other participants present and were audio-recorded. The audio files were transcribed *ad verbatim* from the Ewe or Dangme language into English by the research assistant and three trained transcribers. Where needed, participants were also re-visited to clarify information to enhance the in-depth nature of the data set. A systematic inductive qualitative content analysis [29] was adopted with continual reading and organisation of the data, drawing out analytical and empirical categories as well as themes and triangulating it with observational data. The final stage of the analysis centred around two themes: landownership, and tenancy and their links with livelihoods and investments in sanitation.

## Results

### Characteristics of study communities

#### The fishing community

The community’s members were from the Ewe ethnic group and migrated from the Volta Region to settle here over a century ago. They are, however, still considered an immigrant population serving as ‘caretakers’ of the land, which is owned by the indigenous population of the area – the Osudoku ethnic group. We observed that the community lies within a few miles of a large hydroelectric dam on the Volta River. Their livelihood mainly consists of artisanal fishing (small-scale fishing using traditional methods), supplemented by small-scale tilapia aquaculture, animal husbandry and subsistence farming. A few residents also work for commercial sand mining companies. Respondents reported that fishing is now less profitable due to the fast flowing water released during the periodic opening of the dam’s sluices. Due to periodic flooding from the dam, the land size has also shrunk, which meant that the original fishing families had lower incomes and had shifted occupations.

The community consists of 25 households with approximately 120 people living in small households of an average of 5 people. We saw that their houses are simple with temporary structures made of laterite with thatched roofs, scattered unevenly with farming plots occupying the remaining land space. The community relies on natural water bodies including the Volta River as their main water source. We also noticed that the sanitation coverage of this community is poor with access to only two simple dug-out trench latrines to share between men and women. Latrines have wooden slabs to squat on but no walls and roofs. Children are not allowed to use this facility for fear that they may fall into the trench. For children, therefore open defecation is the norm. The community members constructed the latrines together and replace them approximately every six months when they fill up. Most community members rely on ‘open defecation’ i.e. defecating in the river or on farm plots. One household had invested in a private VIP latrine with wooden walls and aluminium sheet roofing and a ventilation pipe to draw out smell and flies.

#### The farming community

This community is inhabited mainly by indigenes recognized as the sole land owners within the study area. Their entitlement as land owners stems from being the initial settlers and through securing the land from marauding groups centuries ago. Land is traditionally divided among families according to clan affiliation. Over one decade ago, disagreements between members of the autochthon clans within the Area Council over rights to rice farming land generated a serious conflict resulting in fatalities, and widespread destruction of property and displacement of some community members. As part of the conflict, the traditional leadership was denounced. As at the time of the study, we observed that there was no functional traditional authority. The community, together with the fishing and farming communities, therefore rely on the district officials to manage public affairs.

The community consists of 768 individuals living in 162 households. Houses were seen to be of better standards than those of the fishing community, built of cement or laterite walls and with zinc roofing. After Ghana’s independence in 1957, much of the ethnic group’s land was acquired by the government to build a sugarcane factory. In 1996, after the sugar factory ceased operations, several commercial agro-businesses commenced on the ethnic group’s lands, including several rice farms, two banana export companies, a poultry farm and a foreign-owned sand mining factory. Presently, the amount of accessible farming land for indigenous farmers is very limited. However, agro-businesses employ many of the community members and migrant workers of the area who also supplement incomes with petty trading (informal income generation involving small-scale production and/or generation of small inexpensive items).

The community residents mainly depended on water from one public stand pipe while a few households also had a private water connection. Some families also used surface water from the nearby Volta River. The sanitation situation was somewhat better here compared with the fishing community. The majority of the community members depended on two public VIP toilets built around the time of Ghana’s independence. One was still functioning at the time of the study while the other had been slated for demolition. We could see that the community had themselves established two additional dug-out pit latrines at the opposite ends of the community and both were observed to be almost full and ready for excavation. It was also common practice here to resort to the river, canal, and bushes for open defecation. One household had a water closet, and a few others had built VIP latrines.

#### The estate community

This peri-urban community will be referred to as ‘the estate community’. Following the collapse of the sugarcane factory in the early eighties, a large irrigation scheme was established under the government’s agricultural sector to support rice cultivation. The community is made up of 817 people, living in 194 houses which were initially all built by the government to accommodate government and public sector workers of the irrigation project. The current residents are still mainly government workers with the irrigation project and a few teachers and nurses. Some residents supplement their salaries with commercial rice farming, subsistence farming and petty trading.

We noticed that the houses for high income workers are made of cement but accommodation for lower income workers consists of block houses made of laterite with cement plastering. When the infrastructure was established in the 1950s, water and sanitation were of the highest standards with water closets (WCs) and in-house water networks and sanitation connections to a central sewage system. However, there had been no major investments in maintenance over the last four decades. This has resulted in broken down water connections and non-functioning WCs in a large proportion of houses, choked sewages lines and damaged water networks. These faults require large-scale repairs. We noticed that the affected community members have established individual dug-out latrines in their private gardens, with another latrine for the community kindergarten and one shared by the hostel complex residents.

Further information on the different characteristics of the study communities has been provided in Table 1 below.

**Table 1:** Table showing different characteristics of the 3 study communities

Characteristics	Fishing Community	Farming community	Estate Community
Ethnic Composition	Predominantly Ewe ethnic group	Predominantly Ga-Dangme ethnic group	Made of Ewe, Ga-Dangme and Akan ethnic groups
Sanitation provision	2 simple dug-out trench latrines, open defecation	2 public VIP latrines, two additional dug-out pit latrines, 1 water closet, open defecation in the river, canal and bushes	Water closets supplemented with private dug-out pit latrines
Livelihoods	Artisanal fishing, small-scale tilapia aquaculture, animal husbandry, subsistence farming, commercial sand mining	2 banana export companies, rice farms for individual farmers, a poultry farm and a foreign-owned sand mining factory, petty trading	Government workers with the irrigation project, government teachers and nurses; commercial rice farming, subsistence farming and petty trading
Land tenure	Migrant occupiers of the land (caretakers)	Indigenous landowners	Government owned housing

### Main factors affecting sanitation investments

The findings revealed that broadly, land rights under the following categories could be related to investments into sanitation. These included land availability for household toilets; livelihood developments; maintenance of toilet facilities; investments into household toilet facilities, indigenous land rights; incentives for sanitation investments. Also, a second broad theme, housing and tenure security could be associated with sanitation investments and the following categories emerged from the findings: land tenure; incentives for sanitation improvements; accommodation and rental issues; tenant rights and agreements.

#### Influence of land rights on sanitation investments

As illustrated in the community profiles, inadequate or non-functioning sanitation facilities resulted in community members’ sharing overburdened public and low quality private facilities, and use of open defecation as the only alternative. So the question may be asked: why did families endure these conditions and why did they not invest in private toilet facilities? One of the underlying factors correlated with this sanitation situation across the three different study communities is land ownership and land rights.

By the government’s annexation of traditional lands, successive generations within the farming community experienced decreasing size of land for residential housing and farming. Following land losses, the indigenes, as by right, expected compensation from the government including the development of infrastructure, provision of subsidized potable water, reduced electricity tariffs, and increased employment opportunities. A local council official expressed his dissatisfaction with the loss of land and livelihoods thus: *“The government is not taking care of us because by providing land for two dams generating electricity, we should get light at reduced rates. The government has deprived us of our livelihood but we are not gaining anything”*. Two other farming residents also expressed a clear expectation during the in-depth interviews that the government should provide and maintain public latrines. One male said, *“The government is supposed to help….since we have been paying a toilet toll, the district (government) is supposed to maintain the public toilet for us.”*

With the reduced availability of land, four of the farming community members also expressed difficulties finding available sites for building private latrines: *“The buildings are clustered together so where will they get any space to put a toilet?”* (Male Area Council official). A female farming community resident further explained, *“We didn’t build a household toilet because we don’t have a place to do it. We don’t have land here”.* The lack of entitlement to privately owned land and being unable to designate it for sanitation structures thus made communal toilets the only suitable sanitation solution for this community.

In contrast to the farming community, the estate community populated mostly by non-indigenes was observed to have superior housing, including WC facilities. However, according to the traditionally perceived rights to the land and the properties on it, the indigenes feel entitled to take over the properties and sanitary infrastructure on the land.

This has created tension between the indigenes and non-indigene officials as well as community members in the area. A male estate community resident said during his in-depth interview that: *“The indigenes* [from the farming community] *have been complaining that the town is for them. They complain that we are enjoying (relatively improved facilities) in the estate but they are suffering in the town…so we should go to our hometown”*. This was confirmed by a male indigene living in the estate who opined thus: *“Most of the estate workers and residents are not from here so they seem not to care about the sanitation and the environment; they think they are only coming to work here”*. An indigene male Area Council official remarked that the non-indigenes had better standards of housing and amenities such as sanitation, while the original population was not able to claim the lands, the properties on it or the benefits of the development: *“We as Osudokus should consider ourselves first when allocating lands - before we consider non Osudokus - because we are suffering”*. On the contrary, a male non-indigene Area Council official felt that “*the town is expanding towards the estate community. The indigenes should appreciate the estate’s infrastructure since they will also benefit from any improvements*”.

Non-indigene residents of the area are still considered ‘strangers’ by the autochthon population. In-depth interviews suggest that this has resulted in migrating and non-indigene residents being unlikely to invest in permanent houses and permanent sanitation facilities. Following a job transfer or upon retiring, most interviewed non-indigenes said they would leave the area. A female teacher in the estate explained, *“I don’t have a toilet or bathroom and I am really suffering so if I find a better room elsewhere I will go and rent it. I have applied for transfer from the district several times*”. In contrast, a non-indigene male licensed chemical seller who considers himself an indigene attributed his changed status to his marriage to an indigene. He disclosed during the in-depth interview thus: *“I came here as a stranger to do business. Now, I am one of them since I married a woman from here - for security reasons”.* This changed status has increased his willingness to invest in a local business and in a higher standard apartment with a water closet toilet attached to the central sewage system.

The fishing community residents did not own any part of the land. Unlike the indigenous farming community, the fishing immigrant community considered themselves unable to agitate for any community development projects. A fisherman explained: *“The previous government built public toilets for the nearby communities (indigenes) but since we are Ewes on the indigenes’ land, they did not build one for us so we use the (self-made) pit latrine”.* A male community member confirmed this: “*We here are all Ewes on Osu Doku land. Hence, we are like visitors. We’re the only community in the district without electricity and water, and our (self-made) pit latrine is not the best”*.

Despite this resignation, some expectations for improved community development and sanitation have grown from the pledges of aspiring politicians to develop the area: “*We need a public toilet and piped water but the past political leaders didn’t care about us; our new assemblyman is trying to bring us water”* (Male, resident, in-depth interview fishing community area). The elected local government representative further explained that the provision of water and sanitation facilities was central to the community and for leaders, this is linked to their future electoral fortune: *“The district assembly has to come in and give the community water, light and a place of convenience (toilets) because if you don’t do anything for them, they will not vote you into power again”*.

In summary, land rights and the desire to invest in sanitation facilities played out in significantly different ways in the three communities, leaving non-indigenes, temporary residents and people losing land to government development with limited incentives and opportunities to invest in private improved sanitation.

#### Housing, tenure insecurity and sanitation investments

Non-ownership of land was intrinsically linked to issues of property rentals. Thus, issues of housing and insecure tenancy conditions emerged as another cross-cutting issue related to the ability and willingness to invest in private sanitation. We discovered that the growing commercial agricultural businesses had created an increased influx of workers into the study area for short or long-term work stays. Combined with decreasing amounts of lands available for housing, this has aggravated the scarcity of accommodation. We saw that most of the in-migrating farm workers reside in neighbouring farming communities. This demand for housing was described by a male migrant banana farm worker thus: “*In this town, they never built to rent but because of the banana farm they have begun to rent out their property*”. People reportedly hired single rooms for between 8–20 Ghana Cedis per month (approximately $3–$9) at the time of the study with most rooms having no access to private sanitation. One male renter complained: *“There is no bathhouse, no kitchen, nothing; so now I have to go and beg the neighbours to use their bathroom”.*

Generally, respondents renting accommodation clearly communicated their unwillingness towards making a major financial investment into someone else’s property. A male rice farming community resident said *“I am not supposed to use my money for his house. If I leave the house, then it is for the owner”.* Respondents said that tenants risked landlords (or property owners) increasing their rent if a tenant made improvements to raise the housing standards, while refusing to reimburse the money spent by tenants. A female farming community renter said: *“I painted the room myself. I thought it will be deducted from my rent but they said they don’t do it that way”.* This was also the case for improving sanitation facilities: *“If you are in a rented compound house, it is difficult to say you want to build a toilet. Are you going to charge the landlord for the toilet or would you do it for free and leave it behind when you leave the house?”* (Male estate resident). Combined with the fact that most tenancy agreements were fluid and verbal, tenants’ right to negotiate access to sanitation were very limited and gave landlords’ low incentives and no obligations to improve sanitary facilities for tenants.

The problem of motivating landlords to invest in sanitation was a common theme across all communities, but was differently configured in the fishing community. Here, landowners traditionally did not charge tenants (land-occupiers) any rent. This was due to a long-standing traditional and oral land-occupancy agreement between landowners and land occupiers, in which the non-indigenous land-occupiers offer in-kind appreciations to the landowners for being allowed to be care-takers of the land. The fishing community’s headman explained: *“Our ancestors only had to give palm wine and schnapps (alcoholic drinks) as compensation for living on the land since we are taking care of the land for the owners. Our people were looking for a place and they (land owners) gave the land to us”.* This created a situation, where land-occupiers had access to very simple but no-cost accommodation. As in the farming community, this has placed no obligations on or created incentives for the fishing community’s landowners to provide private sanitation for their ‘tenants’.

In the estate community, land tenure was vested in the government, a large dynamic and mainly faceless authority, renting out to government staff. Here, we found out that government officials attributed the poor maintenance of its sanitation structures for tenants to the lack of sufficient funds since central budgetary disbursements to the irrigation project do not cover sanitation improvements. Furthermore, for the estate community, it was observed that the tenants of the government properties are unable to compel the authorities to improve the sanitation given their position as employees. One female resident said this, *“since the project managers have kindly offered us accommodation when others can’t get a place, perhaps there is no money for renovations since not everyone pays their rent”.* A male tenant similarly explained that: *“Some of our toilets are not functioning and even though the irrigation authorities are supposed to maintain them, they too say they don’t have enough funds to buy the needed materials”.* Interviews with officials of the irrigation projects showed that they disagreed and expected tenants to pay for maintenance of sanitation facilities: *“You cannot expect the irrigation project to do all those repairs for the tenants. The tenants can employ someone to do the repairs because a broken down latrine will disturb the occupants more than the irrigation project - since they (tenants) are living in the houses”*- (Mid-level irrigation project worker).

One senior irrigation project worker confirmed this view: *“The rehabilitation of the sewage system is a major investment and since people are not paying much rent, the project cannot afford the cost”*. The rent, which the respondents said was affordable and below market value, was deducted directly from the salaries of the irrigation project employees while other residents had to make individual payments. One irrigation project worker explained, *“For a member of staff, they reduce the rental charges, which are deducted from our salary”*.

Since the government’s local representatives did not own the properties or benefit directly from the rents being charged, there appeared to be little incentive for them to find the resources required for major investments to improve the properties. Our observations in the estate showed that the cost of necessary sanitation renovations was likely to be too high for workers to cover from their salaries, since systemic and large-scale repairs were necessary.

In summary, in-migration and short-term employments, insecure and informal tenureship, and lack of tenancy entitlements and rights were clearly associated with lower tenants’ and landlords’ abilities and willingness to invest in private sanitation – each trying to maximize and secure incomes and livelihoods in a rapidly changing and uncertain labour and housing market.

## Discussion

### Improving sanitation investments by addressing land ownership and land rights

This study shows that investments in sanitation in this area of rural and peri-urban Ghana is clearly related to by security of historically-bound land ownership and rights, indigenous land distribution systems and its inherent conflicts with contemporary commercial interests and developmental projects. These land issues are intertwined with changing livelihoods and have thus created different premises for investing in sanitation.

This and Simon’s study [30] show similar examples of how Ghanaian rural and peri-urban communities on the verge of urbanization are challenged. An increased sale of indigenes’ land for commercial activities and in-migration of ‘foreign’ labour is followed by a decrease of available land and water access and thus unstable livelihoods when depending on farming and fishing. In our study, this challenged environment clearly hampered sanitation investments. Our study has added that this situation is further complicated by traditional land distribution systems, usages and nuances. Adding to previous studies about land regulations and land ownership in Ghana [14, 15] we found that complex power dynamics of politics, ethnicity and ancestral land rights are co-governing sanitation developments [31].

As emphasized in a review by Place across several African settings [32], we also found that increasing commercial land interests are now challenging indigenous systems of land distribution. Boone [33] also reminds us that often governments’ subjugation of ancestral land access within Tanzania and other African countries has resulted in forced in-migration which renders settlers vulnerable if central authorities withdraw protection. We are, therefore, in agreement with Gandy [34] and Simon [30] in highlighting that urban and peri-urban space in Africa is decreasingly spatially homogenous and holistically planned, but fragmented and polarized. This thus leaves many families without rights to land, fragile livelihoods and no abilities to invest in improved housing and sanitation. With these new land dynamics, sanitation, water, sewage and waste policies and systems in Ghana and possibly many other fast urbanizing areas in Africa are in great risk of failing. We argue that land ownership, land rights and urban space planning therefore need to be considered alongside the sanitation agenda, since these may have an influence on the success of sanitation investments.

### Addressing tenancy insecurity and sanitation maintenance responsibilities

This study has demonstrated how changing and unstable livelihoods are also related to housing and tenancy situations and results in low investments into home sanitation facilities. Similar to a study from the nearby Volta Region [35], we have shown that migrant community residents, who have no chance of owning land and constructing private houses, continue to live in dilapidated and temporary housing without incentives for improving private sanitation, even decades after their arrival in the area. Similarly, according to Isunju and others’ 2011 review of the socio-economic factors affecting sanitation provision in rapidly urbanized areas of East Africa, despite widespread demand for sanitation improvements through landlords, this has not resulted in appreciable changes. Here, tenants’ demands are affected by limited mandates, scarcity of suitable housing, and short-term stays [36].

In addressing landowners’ refusal to provide sanitation facilities, one wonders why they should when tenants pay next to nothing for occupying the land? We believe that part of tenants’ unwillingness to invest in the land they occupy may also stem from the fact that Ewe fishermen traditionally tend to be migratory; they literally follow the fish stock. And this explains why the fisherfolk along the coast of Ghana and other West African countries often build temporary structures using coconut tree branches [37, 38]. Who builds sleeping rooms with coconut tree branches and uses cement to build his toilet?

Also, the short-term migrant residents who are living in government housing or in privately rented rooms with no formalized tenancy agreements see no incentives for improving sanitation. Demanding these residents to invest in a household toilet seems infeasible.

This research complements the findings of other studies that suggest that secure and permanent land tenure is important for creating incentives to improve housing and adhere to housing regulations, which mandate the building of household toilets [18, 19, 39]. A paper evaluating the effect of tenure security on urban household sanitation in Senegal furthers this argument by stressing that improving tenure security i.e. land titling or socio-economic status can significantly increase these investments [40]. This study adds that the high demand for basic and affordable housing and influenced by an increase in in-migrating short term-contract workers, combined with landlords’ refusal to provide the financial resources to establish and maintain toilets, are key reasons for the low sanitation investment rate. Further, most of the tenants may be unable or unwilling to pay higher rents to cover a landlord’s sanitation investment expenses. Further, most of the tenants may be unable or unwilling to pay higher rents to cover a landlord’s sanitation investment expenses. While some empirical evidence [41–43] supports the view that tenure legality may not prevent housing investment, since perceived tenure security could itself stimulate investments, both viewpoints agree that legal status improvements greatly improve the resource mobilisation process [44].

One main reason for this lack of sanitation progress was the lack of formalized agreements on sanitation maintenance costs and responsibilities. In the government housing complexes, this has worsened the breakdown of a once admirable sanitation-sewage system and in the farming communities, on-going protests and disagreements over government and private responsibilities have halted all sanitation progress. Across study communities, this resulted in people reverting to using very poor standard make-shift latrines.

As phrased in a recent analysis by The World Bank on sustaining poor-inclusive urban and peri-urban sanitation systems [45], sanitation progress needs clear accountability structures – both upstream towards authorities and downstream towards citizens. It must be emphasized that since the governmental sanitation agencies have no influence over land ownership and distribution, sanitation progress may be hampered without resolving the broader issues of land tenure and security. Hence, clarifying local authorities’ responsibilities in maintaining large sanitation systems and addressing landlords’ responsibilities for providing sanitation to tenants are two potential ways of improving sanitation standards. A third way is to advocate for long-term formalized tenancy contracts for workers. This may release more capital for landlords to maintain facilities while long-term tenants may feel more incentivized to improve and maintain their accommodation and sanitation facilities.

### Limitations of the study

The difficulty of researching land issues especially within an area that has experienced violent ethnic and political unrests is obvious and has probably influenced the degree of community members’ openness to discuss land issues openly. As described in other sanitation studies in Ghana [46], some apprehension was also encountered when speaking openly about issues of defecation and toilets, since this is related to social stigma and general awkwardness. The long term fieldwork in a limited number of communities providing adequate time to build rapport with informants was therefore chosen as the design likely to yield the best quality of data. It must be noted that there is the potential for bias as a result of using referral sampling though efforts were made to ensure the findings and conclusions could be analyzed for their applicability, consistency and truth value [47, 48]. The cultural sensitivities influencing the lack of sanitation within the study communities emerged from the community entry processes, challenges with the informed consent processes, barriers from residents’ expectations from the research and language difficulties. Additional concerns included negotiating the role of gate keepers in the research process as well as reflections on personal positions.

These issues were addressed through the researchers’ knowledge about relevant cultural norms for gaining access into different research settings, considerable fluency in the community’s local languages and continuous critical reflection of observed practices and emerging questions during the research process to gain additional clarity. Other issues involved navigating the conflict between using standard qualitative research approaches and the reality of doing actual fieldwork as both as outsider and insider.

The economic drivers were unfortunately not directly measured during the data collection process. It was difficult to isolate the effect of land tenure from the socio-economic position of the communities which also explains the lack of emphasis on poverty as a key factor influencing investments in sanitation. While recognizing their importance, the study focused on the underlying socio-cultural reasons that influence the lack of sanitation that were not necessary economic.

## Conclusion

Reported findings represent rapidly changing rural and peri-urban communities in the southern parts of Ghana. This study offers valuable insights into how challenges of sanitation investments are related to poor land rights, insecure tenancy and challenged livelihoods across these spatially and socio-economically different communities. It is clear that sanitation policy makers and programmers must address the complexities of local ethnic, political and economic forces controlling land use, land acquisitions, land rights and tenant agreements to facilitate increased household investments into sanitation.

## Abbreviations

CWSA, Community Water and Sanitation Agency; DHRC, Dodowa Health Research Centre; HDSS, Health and Demographic Surveillance System; MDGs, Millennium Development Goals; VIP, Ventilated Improved Pit toilets; WHO, World Health Organization
